# Targeting Cancer Cell Tight Junctions Enhances PLGA-Based Photothermal Sensitizers’ Performance In Vitro and In Vivo

**DOI:** 10.3390/pharmaceutics14010043

**Published:** 2021-12-26

**Authors:** Victoria O. Shipunova, Vera L. Kovalenko, Polina A. Kotelnikova, Anna S. Sogomonyan, Olga N. Shilova, Elena N. Komedchikova, Andrei V. Zvyagin, Maxim P. Nikitin, Sergey M. Deyev

**Affiliations:** 1 Moscow Institute of Physics and Technology, 9 Institutskiy per., 141701 Dolgoprudny, Russia; kovalenko.vl@phystech.edu (V.L.K.); lena-kom08@rambler.ru (E.N.K.); max.nikitin@phystech.edu (M.P.N.); 2 Shemyakin-Ovchinnikov Institute of Bioorganic Chemistry, Russian Academy of Sciences, 16/10 Miklukho-Maklaya St., 117997 Moscow, Russia; kotelnikova@phystech.edu (P.A.K.); annasogomonyan2012@mail.ru (A.S.S.); olchernykh@yandex.ru (O.N.S.); andrei.zvyagin@mq.edu.au (A.V.Z.); biomem@mail.ru (S.M.D.); 3 Department of Nanobiomedicine, Sirius University of Science and Technology, 1 Olympic Ave., 354340 Sochi, Russia

**Keywords:** photothermal therapy, phthalocyanine, SKOVip-kat, Katushka, TurboFP635, JO-4, PLGA, orthotopic tumors, 3D culture, spheroids

## Abstract

The development of non-invasive photothermal therapy (PTT) methods utilizing nanoparticles as sensitizers is one of the most promising directions in modern oncology. Nanoparticles loaded with photothermal dyes are capable of delivering a sufficient amount of a therapeutic substance and releasing it with the desired kinetics in vivo. However, the effectiveness of oncotherapy methods, including PTT, is often limited due to poor penetration of sensitizers into the tumor, especially into solid tumors of epithelial origin characterized by tight cellular junctions. In this work, we synthesized 200 nm nanoparticles from the biocompatible copolymer of lactic and glycolic acid, PLGA, loaded with magnesium phthalocyanine, PLGA/Pht-Mg. The PLGA/Pht-Mg particles under the irradiation with NIR light (808 nm), heat the surrounding solution by 40 °C. The effectiveness of using such particles for cancer cells elimination was demonstrated in 2D culture in vitro and in our original 3D model with multicellular spheroids possessing tight cell contacts. It was shown that the mean inhibitory concentration of such nanoparticles upon light irradiation for 15 min worsens by more than an order of magnitude: IC50 increases from 3 µg/mL for 2D culture vs. 117 µg/mL for 3D culture. However, when using the JO-4 intercellular junction opener protein, which causes a short epithelial–mesenchymal transition and transiently opens intercellular junctions in epithelial cells, the efficiency of nanoparticles in 3D culture was comparable or even outperforming that for 2D (IC50 = 1.9 µg/mL with JO-4). Synergy in the co-administration of PTT nanosensitizers and JO-4 protein was found to retain in vivo using orthotopic tumors of BALB/c mice: we demonstrated that the efficiency in the delivery of such nanoparticles to the tumor is 2.5 times increased when PLGA/Pht-Mg nanoparticles are administered together with JO-4. Thus the targeting the tumor cell junctions can significantly increase the performance of PTT nanosensitizers.

## 1. Introduction

The methods of photodynamic (PDT) and photothermal (PTT) cancer therapy are based on the use of photosensitizers that are accumulated in the tumor and lead to tumor elimination after light irradiation. Upon absorption of light of a certain wavelength, the photosensitizer switches to an excited state, from which it can come back to the ground state either radiatively with fluorescence emission or non-radiatively with the release of thermal energy. Photosensitizers can also react with cell components via electron transfer, which leads to the formation of free radicals or transfer energy to oxygen with the formation of highly reactive singlet oxygen [1]. Thus, photosensitizers can lead to oxidative stress on a cancer cell or perform local hyperthermia. The increased sensitivity of cancer cells to heating up to 41–47 °C underlies the effectiveness of photothermal therapy [2].

The main advantages of PDT and PTT are non-invasiveness and spatial selectivity. The photosensitizer should have minimal toxicity in the dark, efficiently penetrate the tumor and accumulate inside cancer and stromal cells. Thus PDT and PTT therapies can significantly reduce side effects and improve the effectiveness and specificity of cancer treatment. However, photosensitizers used in the clinic today can accumulate in the organism, significantly increasing the photosensitivity of the skin [3]. Many photosensitizers suffer from poor water solubility, low bioavailability, and instability in physiological conditions. Due to these negative effects, the use of PDT is limited in clinical practice, but these difficulties can be overcome by chemical modification, PEGylation, or by photosensitizer encapsulation in nanocarriers of various nature [4,5,6].

However, as well as the above-mentioned limitations, there are additional problems that arise in the development of phototherapy and other cancer treatment methods. In particular, solid tumors of the epithelial origin are characterized by tight intercellular contacts limiting the penetration of active substances deeper than 3–4 layers of cells [7]. Preservation of epithelial tissue intercellular contacts is typical for cancer cells and makes traditional chemotherapies as well as targeted therapies with monoclonal antibodies and supramolecular agents ineffective.

To effectively penetrate the tumor through anatomical barriers and actively diffuse within solid tumors, therapeutic agents must bypass the intercellular contacts that seal the boundaries of normal endothelial cells and intercellular spaces within the tumor. To date, the most promising agents that open up cell contacts are the junction opener proteins (JO) obtained from human adenovirus serotype 3 [8,9,10,11]. Protein JO-1 and its improved variant JO-4 bind to desmoglein 2 on the cell surface, which leads to the activation of MAP-kinases that activate the metalloprotease ADAM17, which breaks down desmoglein of cell contacts [10]. Activation of MAP-kinases leads to transient transdifferentiation of epithelial cells, including a decrease in the expression of adhesion and blocking cell contact proteins, thus solving the problem of the diffusion of drugs within the tumor [9]. Thus, JO-1 and JO-4 induce a partial epithelial–mesenchymal transition (EMT), increasing the permeability of the tumor to high molecular weight compounds and protein molecules, including antibodies [12]. The improvement of drug delivery to tumors using JO proteins has been demonstrated thoroughly for antibodies and chemotherapy drugs, but the effect of JO on nanostructures delivery is poorly understood. It was shown that JO significantly increases bulk tumor accumulation of 35 nm but not 120 nm gold nanoparticles [13] and significantly enhances the efficacy of liposomes loaded with doxorubicin in vivo [8].

Here we describe the synthesis and characterization of biocompatible polymer nanocontainers loaded with magnesium phthalocyanine (Pht-Mg) as effective photothermal sensitizers. We showed their effectiveness when exposed to near-IR light in terms of selective destruction of cancer cells in 2D culture. However, during the transition from 2D tests to 3D cell culture, the effectiveness of such agents decreased by more than 10 times. Nevertheless, the use of a junction opener protein JO-4 led to the efficiency of nanostructures in 3D culture, comparable or outperforming that for 2D culture, and significantly increased the efficiency of accumulation of nanoparticles in orthotopic mouse tumors in vivo.

## 2. Materials and Methods

### 2.1. PLGA Nanoparticle Synthesis

Poly(D, L-lactide-co-glycolide) (RG 858 S, Poly(D,L-lactide-co-glycolide) ester terminated, lactide:glycolide 85:15, Mw 190,000–240,000 Da, Sigma, Darmstadt, Germany) was used as a matrix for the nanoparticle synthesis with the “oil-in-water” microemulsion method. The emulsion was formed by dropping the solution of 40 g/L PLGA and 1 g/L magnesium phthalocyanine (Sigma #402737-1G, Pht-Mg) in chloroform into the 3 mL of 3% PVA (Mowiol^®^ 4-88, Sigma, Darmstadt, Germany) in Milli-Q water with 1 g/L of chitosan oligosaccharide lactate (5 kDa, Sigma, Darmstadt, Germany). Alternatively, manganese(II) phthalocyanine (Sigma 379557-1G), copper(II) phthalocyanine (Sigma #546682-200MG) or 29H,31H-phthalocyanine (Sigma #253103-1G) at 1 g/L were used for nanoparticle synthesis instead of magnesium phthalocyanine. The emulsion was continuously sonicated with the Bandelin Sonopuls HD 2200 (Bandelin, Germany) for 1 min with 25% amplitude of 200 W sonicator. The resulting nanoparticles were washed by triple centrifugation with PBS and finally resuspended in 300 µL of PBS. The final concentration of the particles was determined with air drying at 90 °C.

### 2.2. Scanning Electron Microscopy

Scanning electron microscopy images of as-synthesized PLGA/Pht-Mg nanoparticles were obtained with a MAIA3 Tescan (Tescan, Brno-Kohoutovice, Czech Republic) microscope at an accelerating voltage of 7 kV. Samples of PLGA/Pht-Mg particles in water at 10 µg/mL were air-dried on a silicon wafer on carbon film and analyzed immediately. SEM images were evaluated using ImageJ software to get a particle size distribution.

### 2.3. Photothermal Properties Study in Cell-Free System

A colloidal solution of nanoparticles in phosphate-buffered saline was irradiated using a 1200 mW 808 nm laser in a 2 mL test tube. The temperature change was recorded using a FLIR One Pro (Teledyne FLIR, Santa Barbara, CA, USA) thermal imaging camera. Temperature changes are shown as a function of time with substracted the initial room temperature (usually 23–24 °C).

### 2.4. Fluorescence Spectroscopy

The excitation and emission spectra of PLGA/Pht-Mg nanoparticles at 1 g/L were recorded using an Infinite M1000 Pro microplate reader (Tecan, Grödig, Austria) in 50%: 50% DMSO: H_2_O solution. The excitation spectrum was recorded using the emission wavelength of 750 nm within the 280–740 nm range. The emission spectrum was recorded using the excitation wavelength of 350 nm within the 360–850 nm range.

### 2.5. Cell Culture

SKOVip-kat and EMT6/P cells were cultured in DMEM medium (HyClone, Logan, UT, USA) supplemented with 10% fetal bovine serum (HyClone, Logan, UT, USA) and 2 mM L-glutamine (PanEko, Moscow, Russia). Cells were incubated under a humidified atmosphere with 5% CO_2_ at 37 °C. For the cytotoxicity tests cells SKOVip-ka stably expressing red fluorescent protein Katushka were used. These cells were developed by us earlier [14].

For the cell toxicity studies previously obtained human ovarian cancer cells stably expressing fluorescent protein Katushka (far-red fluorescent protein TurboFP635), SKOVip-kat was used [15]. These cells stably express both Katushka protein in the cytoplasm and transmembrane oncomarker HER2 on the cell surface, thus being an ideal model for the study of therapeutic targeting compounds in vivo. These cells have excitation and emission wavelengths, 588 nm and 635 nm, and are suitable for most in vitro and in vivo imaging devices.

### 2.6. Fluorescence Microscopy

For fluorescent microscopy analysis, cells were seeded on a 96-well plate at 5·10^3^ cells per well in 100 μL of DMEM medium supplemented with 10% FBS and cultured for 10 h. Next, Pht-Mg-loaded PLGA nanoparticles were added to get a final concentration of 10 µg/mL. Cells were incubated with nanoparticles for 1 h or 4 h at 37 °C, washed from non-bound particles, and analyzed with epifluorescent Zeiss microscope at the following conditions: for Katushka protein imaging: excitation filter −560/40 nm, emission filter −630/75 nm; for PLGA/Pht-Mg nanoparticles imaging: excitation filter −595–645 nm, emission filter −670–725 nm.

### 2.7. Cytotoxicity Assay

Cytotoxicity of PLGA/Pht-Mg nanoparticles in 2D cell culture was determined using a resazurin-based toxicity assay. SKOVip-kat cells were incubated with nanoparticles at different concentrations in 200 µL of full phenol red-free DMEM medium in a 2-mL Eppendorf tube for 4 h at 37 °C with 5% CO_2_ and then light irradiated with 808 nm laser for different time intervals. Next, cells were diluted with full medium and seeded on a 96-well plate at 2 × 10^3^ cells per well in 100 μL of DMEM medium supplemented with 10% FBS and cultured for 72 h. Next, wells were washed from non-bound particles, and 100 μL of resazurin solution (13 mg/L in phosphate-buffered saline) was added to each well. Samples were incubated for 3 h at 37 °C in a humidified atmosphere with 5% CO_2_. The fluorescence of each well was measured using the CLARIOstar microplate reader (BMG Labtech, Ortenberg, Germany) at wavelengths of λex = 570 nm, λem = 600 nm. Data are presented as percent from non-treated cells.

### 2.8. 3D Culture

A Form3 3D printer (FormLabs, Somerville, CA, USA) with FormLabs Gray Resin 1 L photopolymer resin (FormLabs, Somerville, CA, USA) was used to create master molds for pouring agarose gel to create microwells for cell growth. One percent agarose in phenol red-free growth DMEM medium (Gibco, Carlsbad, CA, USA) without FBS was used. Spheroids were prepared by dropping SKOVip-kat cell suspension into agarose wells with subsequent cultivation in a 12-well plate (Nunc, Roskilde, Denmark) with DMEM growth medium with 10% FBS grown for 3 days with 5% CO_2_ at 37 °C. The formed spheroids were incubated with nanoparticles with or without subsequent irradiation and analyzed with the Axiovert 200 (Carl Zeiss, Göttingen, Germany) fluorescence microscope. The images of fluorescent spheroids were analyzed using ImageJ software to calculate the total spheroid fluorescence using an “integrated density” parameter.

### 2.9. Proteins Purification

JO-4 and DARP-LoPE were purified as described by us previously [15].

### 2.10. Confocal Microscopy

Three dimensional spheroids were incubated with JO-4 at final concentration of 10 µg/mL for 2 h followed with 4-h incubation of 50 µg/mL of PLGA/Pht-Mg nanoparticles in the full cell culture media. Spheroids were imaged with confocal laser scanning miscroscopy. Confocal microscopy images of spheroids were obtained with a FV3000 laser-scanning confocal microscope (Olympus Optical Co Ltd, Tokyo, Japan) using LUCPLFLN 20× objective (20× magnification, 0.45 numerical aperture) with 640 nm laser and GaAsP detector (500 V) (650–750 nm).

### 2.11. Chemical Conjugation

JO-4 labeled with FITC (JO-4-FITC) was prepared by rapid mixing of 100 µL of JO-4 at 1 g/L in phosphate buffer, pH 7.4 with 10 µL of FITC at 0.5 g/L in DMSO. JO-4 labeled with Cy5.5 (JO-4-Cy5.5) was prepared by rapid mixing of 100 µL of JO-4 at 1 g/L in phosphate buffer, pH 7.4 with 10 µL of Cy5.5-NHS ester at 1.0 g/L in DMSO. The solutions of labeled proteins were incubated for 8 h, and the excess of unreacted FITC and Cy5.5-NHS molecules was removed with Zeba Spin Desalting Columns (7 kDa MWCO) according to the manufacturer’s recommendations.

### 2.12. Flow Cytometry

Spheroids were taken out from agarose molds, disaggregated with trypsin/EDTA solution (0.25%) for 15 min at 5% CO_2_ at 37 °C, washed twice with PBS. For the cell viability assays, cells were stained with propidium iodide at 2.5 µg/mL final concentration 5 min before the analysis. The cell populations were analyzed using BD Accuri C6 (BD) flow cytometer using the excitation laser 488 nm and the emission filter 615/20 nm. For nanoparticle binding assay, cells were analyzed with Novocyte 2000R flow cytometer (Acea Biosciences, San Diego, CA, USA) with an excitation laser of 640 nm and emission filter 675/30 nm.

### 2.13. Photothermal Properties Study In Vitro

A total of 2 × 10^6^ cells were incubated with PLGA/Pht-Mg nanoparticles to get a final concentration of nanoparticles equal to 0.5 g/L in 200 µL in 2-mL eppendorf tube. The samples were incubated for 4 h at 5% CO_2_ at 37 °C. Next, the tube with nanoparticles and cells were incubated at 5% CO_2_ at 37 °C for 4 h and irradiated with 808-nm laser. The thermal imaging was performed using a FLIR One Pro (Teledyne FLIR, Santa Barbara, CA, USA) thermal imaging camera. Temperature changes were recorded using a smartphone and are presented as thermal images of the tubes with cells with or without nanoparticles in dependence on irradiation time.

### 2.14. Tumor-Bearing Mice

Female BALB/c mice of 18–22 g weight were used in the experiments. All procedures with animals were approved by the IBCh RAS Institutional Animal Care and Use Committee. The animals were anesthetized with a mixture of Zoletil (Virbac, Carros, France) and Rometar (Bioveta, Ivanovice na Hané, Czech Republic) at a dose of 25/5 mg/kg. Mice were injected with 4·10^6^ EMT6/P cells in the mammary fat pad to create orthotopic tumors. The tumor size was measured with a caliper using the formula V = width^2^ × length/2.

For bioimaging experiments, when tumor size reached 90–100 mm^3^, mice were i.v. injected with 400 µg of PLGA nanoparticles with or without pre-injection of JO-4 (1 h before nanoparticles at 4 mg/kg dose in 0.15 M NaCl).

### 2.15. Ex Vivo Study

For ex vivo tumor study, mice were i.v. injected with JO-4-Cy5.5 (4 mg/kg dose in 0.15 M NaCl). Next, mice were sacrificed with cervical dislocation and cryosections of tumor tissue were obtained using FSE cryostat (Thermo). Confocal microscopy images of spheroids were obtained with a FV3000 laser-scanning confocal microscope (Olympus Optical Co. Ltd., Tokyo, Japan) using UPLSAPO 40×2 objective (40× magnification, 0.95 numerical aperture) with 640 nm laser And GaAsP detector (500 V) (650–750 nm).

### 2.16. In Vivo Imaging

In vivo imaging was performed with a LumoTrace FLUO bioimaging system (Abisense, Sochi, Russia) as follows: mice were anesthetized 3 h after injection of nanoparticles and imaged with fluorescence excitation at λ_ex_ = 630 nm and 655/40 nm filter.

### 2.17. In Vivo Therapy

For in vivo therapy study, when tumor size reached 90–100 mm^3^, mice were i.v. injected with 400 µg of PLGA nanoparticles with or without pre-injection of JO-4 (1 h before nanoparticles at 4 mg/kg dose in 0.15 M NaCl). Next, the tumor area was irradiated with 808 nm laser (1200 mW) as follows: 5 min irradiation, 15 min pause, 5 min irradiation. The tumor size was measured with a caliper using the formula V = width^2^ × length/2 every one or two days.

## 3. Results

**Figure 1:**
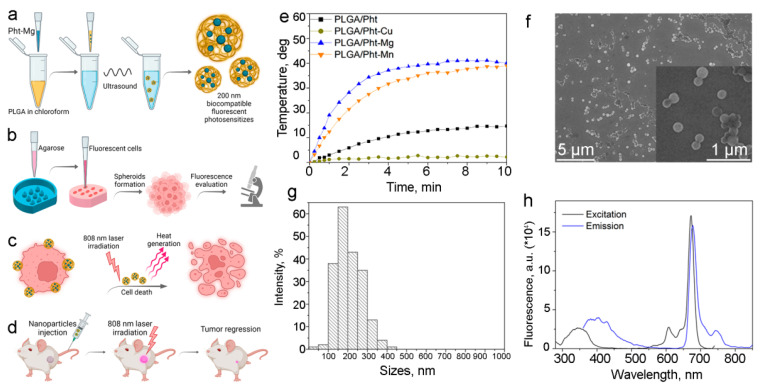
Synthesis and characterization of PLGA-based nanocarriers for photothermal therapy. (**a**) Scheme of nanoparticle synthesis. The mixture of PLGA and Pht-Mg in chloroform is poured into the PVA with chitosan oligosaccharide lactate solution in water and subjected to intensive sonication. (**b**) 3D spheroids formation in agarose gel. Agarose solution was added to the plastic molds and was allowed to solidify at room temperature. Next, a suspension of cells is added to the agarose microwells in order to obtain spheroids on 3–6 days of cultivation. The fluorescence of obtained 3D cell culture is further evaluated using epifluorescent microscopy. (**c**) As obtained 3D spheroids were incubated with PLGA/Pht-Mg nanoparticles followed by 808 nm laser irradiation that leads to the heating of nanoparticles and cell death. (**d**) As-synthesized nanoparticles were used for imaging and treatment of orthotopic tumors in vivo. (**e**) Photothermal properties of PLGA, PLGA/Pht-Mg, PLGA/Pht-Mn, and PLGA/Pht-Cu nanoparticles under the 808 nm light irradiation. (**f**) Scanning electron microscopy images of PLGA nanoparticles. (**g**) Size distribution of PLGA/Pht-Mg nanoparticles, mean size is 208 ± 66 nm. (**h**) Fluorescence excitation and emission spectra of PLGA/Pht-Mg nanoparticles.

The following experimental scheme was designed to assess the effectiveness of PLGA-based nanocarriers. Biocompatible nanoparticles loaded with magnesium phthalocyanine were synthesized by the microemulsion method and used as nanoagents for photothermal therapy and diagnostics in vitro and in vivo (Figure 1a). The resulting nanoparticles were used both for visualization of cancer cells and for their destruction when exposed to light in the transparency window of biological tissue. For the cell toxicity studies previously obtained, human ovarian cancer cells stably expressing fluorescent protein Katushka (far-red fluorescent protein TurboFP635), SKOVip-kat were used [14]. These cells have excitation and emission wavelengths, 588 nm and 635 nm, and are suitable for most in vitro and in vivo imaging devices.

The effect of synthesized particles on 3D multicell spheroids, which were obtained by culturing a cell suspension in agarose microwells, was investigated (Figure 1b). Nanoparticles at various concentrations were added to the formed spheroids and exposed to light with a wavelength of 808 nm (Figure 1c). After the selection the optimal conditions for affecting the spheroids, nanoparticles were i.v. injected into mice for visualization and treatment of orthotopic tumors in vivo.

### 3.1. Synthesis and Characterization of PLGA-Based Photothermal Sensitizers

Nanoparticles of poly-lactide-co-glycolide (PLGA) loaded with magnesium phthalocyanine were synthesized by the microemulsion “oil-in-water” method. The use of PLGA as a drug carrier, attracts the attention of researchers in modern biomedicine, since it provides a stable and controlled release of the compound, thereby reducing side effects [16,17,18,19,20]. PLGA degrades by hydrolysis of its ester linkages in the presence of water and the by-products, lactic acid and glycolic acid, are non-toxic, biocompatible, and rapidly metabolized in the human body.

The photothermal properties of the synthesized nanoparticles were studied under irradiation with an 808 nm laser for 7 min. We tested different NIR dyes to find the optimal for the best performance as a photothermal sensitizer. Among the various NIR dyes tested in this work, including phthalocyanines, magnesium phthalocyanine proved to be the most effective agent for photothermal therapy. Namely, we synthesized PLGA nanoparticles loaded with phthalocyanine (PLGA/Pht), magnesium phthalocyanine (PLGA/Pht-Mg), copper (II) phthalocyanine (PLGA/Pht-Cu), and manganese (II) phthalocyanine (PLGA/Pht-Mn) and tested their photothermal properties (Figure 1d). We showed that the most effective in terms of energy transfer to heat under the 808 nm light irradiation is PLGA nanoparticles loaded with magnesium phthalocyanine (PLGA/Pht-Mg). It was found that the solution of nanoparticles is capable of effectively generating heat energy (Figure 1g). Namely, we have shown that the solution with nanoparticles at a concentration of 1 g/L heats up to ΔT = 40 °C for 3 min and reaches a plateau. Moreover, we have shown that repeated heating of nanoparticles up to three times leads to the same heating of the solution, which indicates that nanoparticles do not degrade and can be used for repeated irradiation to achieve the desired effect.

As obtained nanoparticles PLGA/Pht-Mg were characterized by scanning electron microscopy (Figure 1f). According to the results of SEM image processing, the particles possess a spherical shape and the size of PLGA/Pht-Mg particles is 208 ± 66 nm (Figure 1g).

The fluorescence properties of the synthesized nanoparticles were studied using the fluorescence spectroscopy method. Data presented in Figure 1h confirm that PLGA/Pht-Mg nanoparticles are suitable for fluorescence detection in NIR region, e.g., under the excitation with 640 nm laser as the most commonly used in various visualization devices.

### 3.2. PLGA/Pht-Mg Nanoparticles Interaction with Cells in 2D Cell Culture

**Figure 2:**
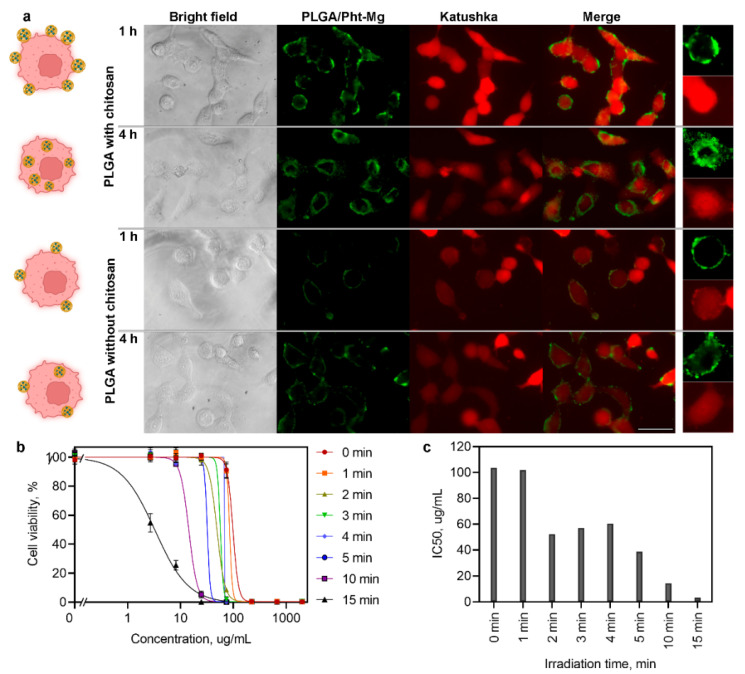
PLGA nanoparticles loaded with Pht-Mg and coated with chitosan oligosaccharide lactate effectively penetrate the cell membrane and possess cytotoxic properties under the 808 nm light irradiation. (**a**) Fluorescence microscopy study of PLGA/Pht-Mg nanoparticles interaction with cells. SKOVip-Kat cells were incubated with PLGA/Pht-Mg particles loaded with Pht-Mg with or without chitosan coating during the synthesis. Cells were imaged 1 h and 4 h after the incubation. Images were taken in a bright field, in the fluorescence channel corresponding to the PLGA/Pht-Mg fluorescence (excitation filter −560/40 nm, emission filter −630/75 nm) and Katushka fluorescence (excitation filter −560/40 nm, emission filter −630/75 nm). Scale bar, 20 µm. (**b**) Light-induced photothermal toxicity of PLGA/Pht-Mg particles for SKOVip-kat cells. Cells were incubated with PLGA/Pht-Mg and irradiated with an 808 nm laser. Cell viability was evaluated 72 h after the irradiation in dependence on particle concentration and irradiation time. (**c**) IC50 dependence on irradiation time for 72 h viability assay.

The fluorescence microscopy study was carried out to assess the effectiveness of synthesized nanoparticles for cell labeling. SKOVip-kat cells were seeded on 96-well plate, incubated with nanoparticles for 1 h or 4 h, and analyzed with an epifluorescent microscope. We showed that under the excitation with 595–645 nm filter and with emission filter of 670–725 nm particles can be effectively used for imaging purposes (Figure 2a). Data presented in Figure 2a demonstrate that after 1 h of incubation the particles are preferably presented outside the cells; however, after 4 h of incubation at 37 °C particles are intensively accumulated in cells.

Moreover, the surface properties of nanoparticles can be effectively used for fine-tuning the particle penetration into cells. To enhance the saturation of cell surface with PLGA/Pht-Mg and PLGA/Pht-Mg nanoparticles accumulation in cells, the surface of particles was modified with chitosan oligosaccharide lactate during the synthesis process (Figure 1a). We showed that using equal experimental conditions, the accumulation of chitosan-coated nanoparticles is more effective in comparison with pristine PLGA (Figure 2a).

### 3.3. Light-Induced Phototoxicity of PLGA Nanoparticles In Vitro

According to the results of fluorescence microscopy, 4 h is enough for the penetration of the main portion of particles into cells. For light-induced cytotoxicity tests, SKOVip-kat cells were incubated with PLGA/Pht-Mg nanoparticles for 4 h and irradiated with 808 nm laser for 1, 2, 3, 4, 5, 10, and 15 min. The cytotoxicity was assessed 72 h later using the resazurin-based cytotoxicity test.

We have shown that the cytotoxicity of nanoparticles was of a concentration-dependent manner and significantly increased when the nanoparticles were heated by a laser. At the same time, the effect of the laser on cells without particles did not have a cytotoxic effect. We showed that IC50 of PLGA/Pht-Mg without irradiation is equal to 98 µg/mL (Figure 2c). However, when cells with particles were irradiated with light for, e.g., 5 and 15 min, the IC50 value significantly decreases—up to 32 and 3 µg/mL, respectively.

### 3.4. 3D Culture

Since the efficacy of both low molecular weight compounds and supramolecular structures differs significantly within in vitro and in vivo models, 3D spheroids are often used as an intermediate step as a more relevant tool for studying drug efficacy [21,22,23,24,25]. In particular, in spheroids, cells form tight intercellular contacts, which are often the main reason for the poor penetration of therapeutic substances into the tumor.

To test the PLGA/Pht-Mg nanoparticles’ efficacy in 3D culture, we developed the 3D cultivation method based on cells culturing in agarose microwells. The method is based on pouring agarose into microwells of a master-mold and subsequent cell cultivation in the obtained agarose microwells.

The master mold that was designed using 3D-printing technology schematically illustrated in Figure 1b. The .stl file for 3D-printing can be uploaded as Supporting File 2 and used for the printing of the plastic master-mold for agarose pouring.

Agarose solution was poured onto the master mold and then left to solidify at room temperature under sterile conditions resulting in the formation of nine 2.3 × 3.3 mm U-shaped microchambers for the cell culture. The suspension of fluorescent SKOVip-kat cells was then added to the wells leading to the formation of spheroids with tight junctions at 3–6 days of cultivation.

The developed method presents a universal tool for 3D culturing of mammalian cancer cells based on reusable molds for agarose, which allows the reproducible formation of multicellular spheroids with tight contacts. The formation of dense cell spheroids in the wells of an agarose gel allows both real-time bright-field and fluorescent visualization, since agarose is an optically transparent material, and the analysis of spheroids separately when they are removed from the molds with a 200 μL pipette tip.

**Figure 3:**
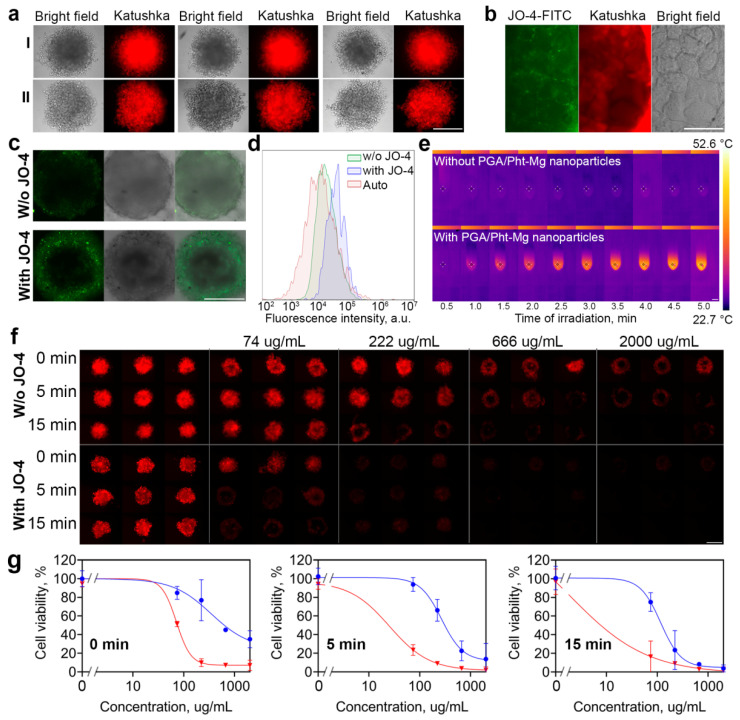
JO-4 enhances the therapeutic and diagnostic capabilities of polymer PLGA-based photothermal sensitizers in vitro. (**a**) Bright-field and fluorescent images of 3D cell spheroids based on SKOVip-Kat cells cultured in agarose molds for 9 days before (I) and after (II) the removal from agarose microwells. Scale bar, 250 µm. (**b**) JO-4 conjugated with FITC interacts with the tight junctions of SKOVip-Kat cells. Scale bar, 20 µm. (**c**) Confocal microscopy images of 3D spheroids labeled with PLGA/Pht-Mg nanoparticles with or without preincubation with JO-4 protein. Images are presented in the fluorescence channel corresponding to the fluorescence of Pht-Mg (excitation 640 nm, emission 660–760 nm, the image is pseudocolored with green color), in the bright field and as a merged image of bright field and fluorescence. Scale bar, 250 µm. (**d**) Flow cytometry assay on evaluation of PLGA/Pht-Mg labeling of cells in spheroids. Spheroids were labeled with PLGA/Pht-Mg with or without preincubation with JO-4 protein, disaggregated with trypsin solution and analyzed with flow cytometry (excitation laser 640 nm, emission filter 675/30 nm). (**e**) Thermal imaging of cell suspension with or without PLGA/Pht-Mg nanoparticles under the irradiation with 808 nm laser with FLIR One camera. Scale bar, 1 cm. (**f**) Epifluorescent images of 3D spheroids of SKOVip-Kat cells incubated with PLGA/Pht-Mg nanoparticles loaded with Pht-Mg and irradiated with NIR light for 0, 5, and 15 min. Scale bar, 250 µm. (**g**) Fluorescence-based cytotoxicity curves for SKOVip-kat spheroids incubated with PLGA/Pht-Mg and irradiated for 0, 5, or 15 min with or without pre-incubation with JO-4 protein.

Data presented in Figure 3a demonstrate the formation of reproducible spheroids from SKOVip-kat cells. Moreover, the spheroids retain their integrity after the removal from agarose microchambers thus confirming that as-obtained 3D cell structures do possess tight junctions. This fact was also confirmed using the 2 mM EDTA solution for the spheroid disaggregation. We noted that 2 mM EDTA which is usually used for the detachment of the SKOVip-kat cells from the plastic surface is absolutely ineffective even after 3 h of incubation for spheroid disaggregation: the spheroids retain their integrity after the incubation. The spheroid can only be disaggregated using concentrated trypsin (0.25%) solution for 15–20 min.

Thus obtained spheroids can be used for further research, for example, for the analysis by flow cytometry after disaggregation, or further cultivation in more complex systems, for example, including cells of the stromal tumor microenvironment.

We showed that the fluorescence intensity calculation using image processing is an adequate tool for the study of cytotoxicity of different compounds in 3D culture in the real-time mode without affecting cell viability and the fluorescence intensity calculation was used in further tests (Figure S1).

### 3.5. Cytotoxicity of PLGA-Based Nanocarriers in 3D Culture

As obtained spheroids were used for evaluating the effectiveness of PLGA carriers in 3D culture mimicking solid tumors.

For the experiment, concentrations within the 74–2000 µg/mL and irradiation time of 0, 5, and 15 min were selected as most clearly reflecting the cell response on incubation with nanoparticles and light irradiation according to 2D cytotoxicity test. Spheroids were overnight incubated with nanoparticles followed by light irradiation and the fluorescence intensity analysis reflecting the cells’ viability was performed on 6 day of cultivation.

Data presented in Figure 3f,g demonstrate that although the cytotoxicity of PLGA/Pht-Mg nanoparticles is quite similar to 2D cytotoxicity tests, much higher doses are required to achieve the desired cell death. For instance, the IC50 of pristine PLGA/Pht-Mg increased to 3.5 times in 3D culture (98 µg/mL for 2D vs. 347 µg/mL for 3D) and to 8.4 times (32 µg/mL for 2D vs. 269 µg/mL for 3D) for 5 min-irradiation and 39 times (3 µg/mL for 2D vs. 117 µg/mL for 3D) for 15 min-irradiation.

To enhance the efficacy of PLGA/Pht-Mg photothermal sensitizers, we used JO-4 protein that induces the short EMT thus allowing nanoparticles to pass through tight cells junctions. JO-4 was added to cells 1 h before the nanoparticle addition and the cytotoxicity test was performed in the same way as for without JO-4. We confirmed that JO-4 conjugated with FITC does interact with the tight junctions of SKOVip-Kat cells when 2D cell culture reaches confluent monolayer (Figure 3b).

Using the laser confocal scanning microscopy, we confirmed that JO-4 indeed enhances the permeability of fluorescent PLGA/Pht-Mg nanoparticles inside the 3D spheroids (Figure 3c). These data are accompanied by flow cytometry assay data (Figure 3d) performed on cells from disaggregated spheroids incubated with PLGA/Pht-Mg nanoparticles with or without pre-incubation with JO-4 protein. Data presented in Figure 3d confirm that JO-4 enhances the particle accumulation in the total cell population within the spheroid. Thus, the cell tight junctions targeting with JO-4 protein and cancer cell heating with PLGA/Pht-Mg sensitizers were used for selective destruction of cancer cells in spheroids. Before that, the retaining of hyperthermic properties of PLGA/Pht-Mg particles after cell binding was confirmed with photothermal imaging using FLIR One pro camera (Figure 3e).

Data shown in Figure 3f,g confirm that the approach is very effective leading to nanoparticle performance increase in 3D culture as it is in 2D culture. Indeed, the IC50 value calculated for viability curve of PLGA/Pht-Mg particles without irradiation was found to be 72 µg/mL (comparable to 98 µg/mL for 2D), 25 µg/mL for 5 min irradiation (comparable to 32 µg/mL for 2D) and 1.9 µg/mL for 15 min irradiation (comparable to 3 µg/mL for 2D).

### 3.6. JO-4 Enhances Nanoparticle Accumulation in Tumor In Vivo

As-obtained nanoparticle PLGA/Pht-Mg performance was assessed in vivo using orthotopic mouse mammary tumors. BALB/c mice were injected with EMT6/P cells to get tumors in the mammary fat pad.

**Figure 4:**
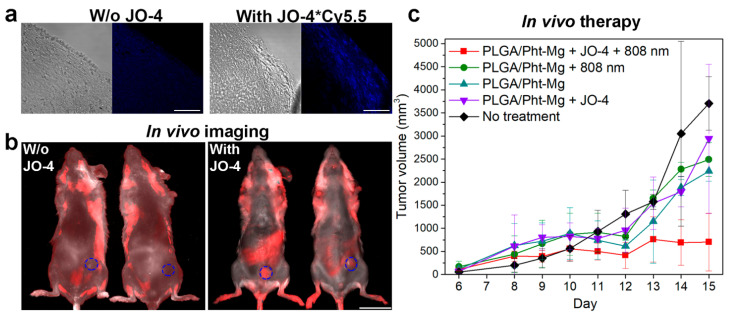
Targeting cancer cell junctions with JO-4 enhances the therapeutic and diagnostic capabilities of polymer PLGA-based photothermal sensitizers in vivo. (**a**) Cryosections of tumor tissue demonstrate the Cy5.5-labeled JO-4 accumulation in the tumor. Confocal laser scanning microscopy images are presented in the fluorescence channel corresponding to the Cy5.5 fluorescence (excitation laser 640 nm, emission 660–760 nm) and in the bright field. Scale bars, 100 µm. (**b**) In vivo living imaging of BALB/c mice with orthotopic tumors i.v. injected with PLGA/Pht-Mg particles with or without pre-injection of JO-4 in the fluorescence channel corresponding to the nanoparticle’s fluorescence. Scale bar, 2 cm. (**c**) Tumor growth dynamics under the treatment with PLGA/Pht-Mg particles with or without JO-4 pre-treatment and with or without 808 nm laser irradiation. Data are presented as mean ± s.d.

Mice were i.v. injected with JO-4-Cy5.5 and cryosections of tumor tissues were analyzed with confocal microscopy thus confirming the accumulation of fluorescently labeled JO-4-Cy5.5 protein in the upper layers of the tumor (Figure 4a).

For bioimaging tests, mice were inoculated with 400 µg of PLGA/Pht-Mg nanoparticles with or without pre-injection of JO-4 and 4 h later visualized with LumoTrace imaging system (Figure 4b) using the excitation diodes of 630 nm and 655 nm emission filters. Data shown in Figure 4b confirm the effective PLGA/Pht-Mg particles accumulation in the tumor site (blue circled area) only in the case when JO-4 was pre-injected. The fluorescence intensity calculations showed that using the pre-injection of JO-4 prior to nanoparticles leads to the tumor fluorescence intensity increase up to 2.5 times (2.49 × 10^6^ vs. 9.86 × 10^5^ a.u.).

### 3.7. JO-4 Enhances the Therapeutic Capabilities of PLGA-Based Nanosensitizers In Vivo

Finally, we evaluated the therapeutic capabilities of PLGA/Pht-Mg particles in vivo using their photothermal properties. Mice were i.v. injected with 400 µg of PLGA/Pht-Mg nanoparticles with or without pre-injection of JO-4 and irradiated with 808 nm laser by two repeated cycles (5 min each). The dynamics of tumors’ growth recorded for two weeks are presented in Figure 4c. Data in Figure 4c confirm that the affecting the tumor with PLGA/Pht-Mg is effective only in the case of pre-injecting with JO-4 and 808 nm light irradiation; the tumor growth inhibition at 15 day calculated as %TGI = (1 − {Tt/T0/Ct/C0}/1 − {C0/Ct}) × 100 where Tt = median tumor volume of treated at time t, T0 = median tumor volume of treated at time 0, Ct = median tumor volume of control at time t and C0 = median tumor volume of control at time 0 was found to be equal to TGI = 92%.

## 4. Discussion

A large number of photosensitizers require excitation with visible light, and the penetration into deep tissues, especially into solid tumors, is limited. The use of near-infrared wavelengths allows deeper penetration into biological tissues and has less effect on endogenous chromophores such as hemoglobin. Optimal irradiation is provided in the so-called biological “transparency window” of 650–950 nm [26]. Longer wavelength ranges lying beyond 1000 nm (the maximum absorption of water) are also reported [27,28]. It should be noted that light in the visible and near-IR ranges is relatively safe for surrounding tissues in a much wider range of irradiation energy used, compared to, e.g., radiation therapy of tumors.

Phthalocyanine-based compounds weakly absorb light at 400–600 nm and practically do not lead to photosensitivity of the skin [29]. However, their ability to absorb light and fluorescence at 650–800 nm and generate singlet oxygen can be used to destroy both skin and deep tumors [30,31,32,33]. The photodynamic and photothermal properties of phthalocyanines depend on their structure. The efficiency of heating or generation of ROS depends on the ion at the base of the complex (for example, heavy metal ions can increase the generation of ROS), and the chemical modification can increase the solubility of phthalocyanines or change the excitation wavelength of their photosensitizing properties [34,35]. Due to the high stability of the phthalocyanine molecules [35], they can be used for repeated cycles of irradiation without any additional injections, in contrast to, e.g., well-studied indocyanine green photothermal sensitizer.

A number of drugs based on phthalocyanine and its derivatives are at various stages of clinical trials and even approved in the clinic thus confirming their efficiency for PDT and PTT. The phototherapy drugs used include hydroxyaluminium trisulfophthalocyanine (Photosens), which has been tested on a variety of tumor types, including eye and eyelid cancer [36], bladder cancer and cervical cancer [37,38,39]. A number of drugs based on phthalocyanine derivatives have undergone different stages of clinical trials and include zinc phthalocyanine derivatives (CGP55847 [40] and Photocyanine [41]), silicon (Pc 4 [42]). Currently, a large number of phthalocyanine-based drugs are being developed for photodynamic [43] (Li et al. 2018) and photothermal therapy [44,45] cancer.

In this work, nanoparticles based on magnesium phthalocyanines were obtained, which are effectively heated when irradiated with light of a wavelength of 808 nm. We have shown that such particles, when stabilized by chitosan oligosaccharide lactate, are selectively internalized into cells and can be effectively used for laser-induced cell death in 2D in vitro culture.

However, when 3D cellular spheroids were used, the efficiency of these particles decreased significantly more than an order of magnitude in terms of IC50. To meet the challenge and increase the nanoparticle performance, especially their penetration into tight multicellular structures, we used the combination of PLGA-based photothermal sensitizers and the protein JO-4 that weakened intercellular contacts by activating metalloprotease ADAM17, which breaks down desmoglein of cell contacts and increased tissue permeability. After the binding to desmoglein 2, these proteins cause temporary transdifferentiation of cells, a partial epithelial–mesenchymal transition (EMT). JO-4 was obtained by screening mutant variants and has a higher affinity for desmoglein-2 than JO-1. JO proteins are able to induce the opening of cell contacts in the normal stratified epithelium; however, JO-1 has been shown to preferentially act on intercellular contacts of tumors and does not cause serious side effects at therapeutic doses [9,11,12]. This was confirmed for JO-1 in xenograft models [9,12], the safety of JO-4 has also been shown in macaques [8]. Thus, JO proteins can be used to overcome anatomical barriers and improve the diffusion of therapeutic agents within a tumor. At the same time, the change in the cellular phenotype of the tumor is temporary, and long-term experiments have shown that the use of JO in vivo does not increase the likelihood of metastases [11].

Of course, the application of photothermal therapy with photothermal nanosensitizers and NIR light irradiation has serious limitations regarding light penetration into tissues. However, light sources in the 800–900 nm wavelength range are the most effective for PTT from UV-Vis-NIR range since the light penetration depth is about 4–5 mm in this range in comparison with, e.g., 0.5 mm for blue light. The described method of PTT can be significantly improved with the use of invasive light sources or in the combination with chemotherapy or radiotherapy that does not require external light sources. Namely, the PLGA/Pht-Mg sensitizers can be additionally loaded with chemotherapeutic drugs—doxorubicin or paclitaxel and used in co-administration with junction opener proteins to perform better for oncotherapy.

Previously the effectiveness of the JO-4 protein in relation to nanoparticles was studied in relation to PEGylated liposomes and gold nanoparticles only. Thus, the effect of this protein on enhancing the effectiveness of liposomes loaded with doxorubicin in vivo [8] was demonstrated, and it was shown that JO protein significantly increases bulk tumor accumulation of 35 nm but not 120 nm gold nanoparticles [13]. Here we show that targeting tight junctions is effective for quite large 200 nm polymer nanoparticles acting as sensitizers of PTT thus opening up new possibilities in cancer treatment using polymer-based nano-cargo. However, the systemic toxicity of such treatment still remains an open question and should be thoroughly investigated after this proof-of-concept study. Nevertheless, based on previous studies [8], we believe that this method is safe for use in oncology in combination with the use of nanoparticles. Previously, when studying the combination of Doxil and JO-4 in healthy non-human primates the non-significant effects regarding the systemic toxicity were observed: no changes in animal health/behavior were observed; histological analysis revealed mild gastro-enterocolitis, the hematological analysis revealed mild lymphopenia on day 1, and thrombocytopenia at days 1, 2, and 3 after the injection. A mild transaminitis was observed on day 1 after JO-4 injection which could be related to residual bacterial endotoxin in the JO-4 preparation. No significant damage of liver, kidney and spleen was observed. When combined with Doxil, analysis of clinical symptoms and blood parameters did not show remarkable signs of toxicity. Overall, the study (Richter et al., 2015) did not show critical JO-4-related toxicity or an increase of Doxil-related side effects, thus proving the clinical safety of the proposed method for the use in combination with nanomedications [8].

## 5. Conclusions

Despite the significant progress achieved in the treatment of oncological diseases in recent decades, the treatment of solid tumors of epithelial origin possessing intercellular contacts is still a serious problem. Considerable efforts are being made to enhance the efficacy of nanoagents of different origins, e.g., aimed at extending their bloodstream circulation [46,47] or changing their biodistribution [48]. Nevertheless, tight intercellular contacts typical for cancer cells make both traditional chemotherapies and therapy with monoclonal antibodies and supramolecular agents ineffective. Therefore, the active targeting of tumor cell contacts in the development of new and improvement of existing approaches to the therapy of solid tumors is one of the most urgent areas of modern biomedicine.

Here we show that the co-administration of a junction opener protein JO-4 and quite large 200 nm PLGA nanoparticles loaded with magnesium phthalocyanine is an effective method both for therapeutic purposes and for the diagnosis of solid tumors. The developed technology can also be used for other types of polymer nanoparticles acting as PTT or PDT sensitizers.

Since several PLGA-based non-targeted drugs formulations are already entered the clinic, e.g., Lupron Depot (Abbvie Endocrine Inc., North Chicago, IL, USA) and Trelstar (Allergan Sales Inc., Madison, NJ, USA) for prostate cancer treatment, the described technique can probably be used for already approved micro- and nanoparticles for improving their efficiency. We believe that the co-administration of agents targeting tight tumor contacts together with therapeutic nanoparticles can significantly improve both the quality of diagnosis and therapy of solid, including metastatic, tumors.

## Data Availability

All data are presented within the manuscript and supporting information.

## Supplementary Materials

The following are available online at https://www.mdpi.com/article/10.3390/pharmaceutics14010043/s1, Supplementary Note 1: Cytotoxicity tests in 3D in vitro culture, Figure S1: Comparison of cytotoxicity tests for assessing the efficacy of anticancer drugs in 3D culture with multicellular spheroids possessing tight contacts.
