# Supplementary material for: Targeting Cancer Cell Tight Junctions Enhances PLGA-Based Photothermal Sensitizers’ Performance In Vitro and In Vivo
Source: Pharmaceutics. 2021 Dec 26;14(1):43. doi: 10.3390/pharmaceutics14010043 (PMC8778343; doi:10.3390/pharmaceutics14010043)
Supplement: Supplementary file 1 [file pharmaceutics-14-00043-s001.zip › pharmaceutics-1500485-supplementary.pdf]

pharmaceutics

Supplementary Materials: Targeting Cancer Cell Tight Junctions Enhances PLGA-Based Photothermal Sensitizers' Performance In Vitro and In Vivo

Victoria O. Shipunova, Vera L. Kovalenko, Polina A. Kotelnikova, Anna S. Sogomonyan, Olga N. Shilova, Elena N. Komedchikova, Andrei V. Zvyagin, Maxim P. Nikitin and Sergey M. Deyev

Supplementary Note 1. Cytotoxicity tests in 3D *in vitro* culture.

To assess the cytotoxicity of the synthesized nanoparticles, we used the method of processing the fluorescent images of spheroids corresponding to the fluorescence of the Katushka protein that reflects the viability of cells inside the 3D multicellular spheroids. To confirm the validity of the chosen method, we compared three methods for studying the cytotoxicity of compounds using the well-studied targeted immunotoxin DARP-LoPE, obtained by us previously [1–4].

Spheroids were incubated with three concentrations of DARP-LoPE (0.038 nM, 0.38 nM, and 3.8 nM) for 6 days and the untreated spheroids served as control. Next, the cytotoxicity of DARP-LoPE was assessed using the following three methods:

1) Resazurin-based test. Spheroids in agarose molds were incubated with resazurin in PBS (at a final concentration of 13 μg/L) for 24 h and the fluorescence of 100 μL of the solution was measured using the Infinite 1000 Pro (Tecan, Austria) microplate reader at wavelengths of λex = 570 nm, λem = 600 nm. Data are presented as percent from non-treated spheroids.
2) The images of fluorescent spheroids were analyzed using ImageJ software to calculate the total spheroid fluorescence using an "integrated density" parameter with substracted background intensity.
3) Flow cytometry assay was performed to assess the cytotoxicity of DARP-LoPE. Spheroids were taken out from agarose molds, disaggregated with trypsin/EDTA solution, and stained with propidium iodide at 2.5 μg/mL final concentration 5 min before the analysis. The cell populations were analyzed using BD Accuri C6 flow cytometer using the excitation laser 488 nm and the emission filter 615/20 nm.

Data presented in Fig. S1 confirm that the described three methods are in good accordance for cell viability study. The calculated Pearson correlation coefficient for methods 1) and 2) is equal to 0.946 and for methods 2) and 3) is 0.934, thus confirming the validity of all three methods of cytotoxicity assessment.

However, fluorescence quantification using image processing with ImageJ software is much more relevant for 3D culture study since it does not require any sample processing (like cell disaggregation or incubation with different substances, e.g., resazurin) and is performed in real-time mode and can be used for multiple analysis of the single sample, e.g. analysis of viability on 3,6,9 and 12 days of incubation or subsequent analysis with other methods.

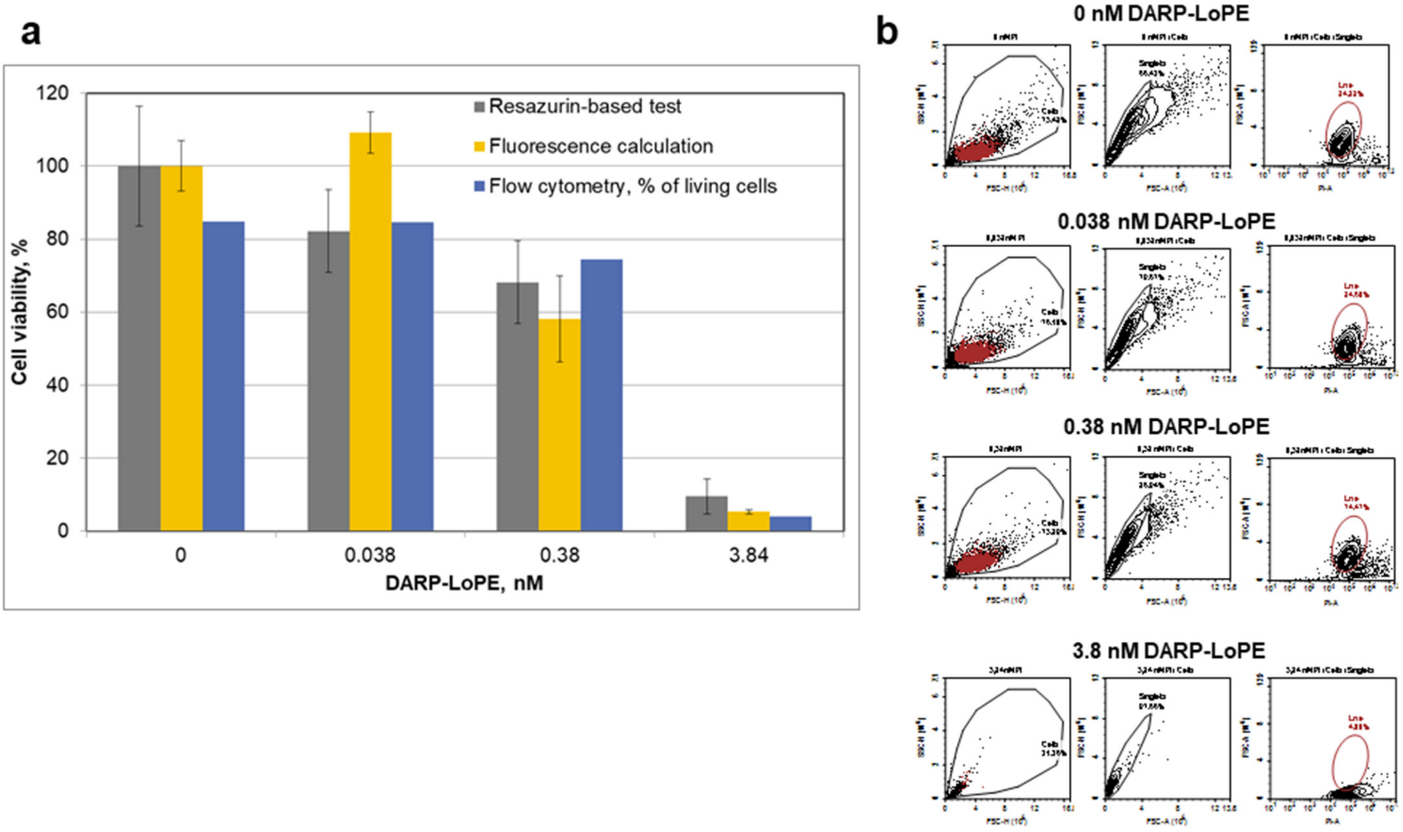

Figure S1. Comparison of cytotoxicity tests for assessing the efficacy of anticancer drugs in 3D culture with multicellular spheroids possessing tight contacts. (**a**) Comparison of the resazurin-based test, fluorescence calculation test, and flow cytometry assay for assessing the cytotoxicity of DARP-LoPE targeted immunotoxin. (**b**) Flow cytometry dot plots reflecting the cell population viability after exposure to DARP-LoPE immunotoxin. The cell population was identified using FSC/SSC dot plots, followed by singlet cells identification with FSC-A/FSC-H dot plot with eventual assessing the percentage of living cells using PI staining in PI/FSC-A dot plot.

References

1. Proshkina, G.M.; Kiseleva, D.V.; Shilova, O.N.; Ryabova, A.V.; Shramova, E.I.; Stremovskiy, O.A.; Deyev, S.M. Bifunctional Toxin DARP-LoPE Based on the Her2-Specific Innovative Module of a Non-Immunoglobulin Scaffold as a Promising Agent for Theranostics. *Mol Biol* **2017**, *51*, 865–873, doi:10.1134/S0026893317060140.
2. Shipunova, V.O.; Komedchikova, E.N.; Kotelnikova, P.A.; Zelepukin, I.V.; Schulga, A.A.; Proshkina, G.M.; Shramova, E.I.; Kutscher, H.L.; Telegin, G.B.; Kabashin, A.V.; et al. Dual Regioselective Targeting the Same Receptor in Nanoparticle-Mediated Combination Immuno/Chemotherapy for Enhanced Image-Guided Cancer Treatment. *ACS Nano* **2020**, *14*, 12781–12795, doi:10.1021/acsnano.0c03421.
3. Sokolova, E.; Kutova, O.; Grishina, A.; Pospelov, A.; Guryev, E.; Schulga, A.; Deyev, S.; Balalaeva, I. Penetration Efficiency of Antitumor Agents in Ovarian Cancer Spheroids: The Case of Recombinant Targeted Toxin DARPin-LoPE and the Chemotherapy Drug, Doxorubicin. *Pharmaceutics* **2019**, *11*, doi:10.3390/pharmaceutics11050219.
4. Shramova, E.; Proshkina, G.; Shipunova, V.; Ryabova, A.; Kamyshinsky, R.; Konevega, A.; Schulga, A.; Konovalova, E.; Telegin, G.; Deyev, S. Dual Targeting of Cancer Cells with DARPin-Based Toxins for Overcoming Tumor Escape. *Cancers (Basel)* **2020**, *12*, doi:10.3390/cancers12103014.
